# Effect of 0.9% NaCl compared to plasma-lyte on biomarkers of kidney injury, sodium excretion and tubular transport proteins in patients undergoing primary uncemented hip replacement – a randomized trial

**DOI:** 10.1186/s12882-021-02310-4

**Published:** 2021-03-26

**Authors:** A. M. Østergaard, A. N. Jørgensen, S. Bøvling, N. P. Ekeløf, F. H. Mose, J. N. Bech

**Affiliations:** 1 University Clinic in Nephrology and Hypertension and University of Aarhus, Gødstrup Hospital, Laegaardvej 12, 7500 Holstebro, Denmark; 2 Department of Orthopaedic Surgery, Gødstrup Hospital, Holstebro, Denmark; 3 Department of Anaesthesiology, Gødstrup Hospital, Holstebro, Denmark

**Keywords:** Isotonic saline, Hyperchloremic acidosis, Acute kidney injury, NGAL, KIM-1

## Abstract

**Background:**

Isotonic saline (IS) is widely used to secure perioperative cardiovascular stability. However, the high amount of chloride in IS can induce hyperchloremic acidosis. Therefore, IS is suspected to increase the risk of acute kidney injury (AKI). Biomarkers may have potential as indicators.

**Methods:**

In a double-blinded, placebo-controlled study, 38 patients undergoing primary uncemented hip replacement were randomized to IS or PlasmaLyte (PL). Infusion was given during surgery as 15 ml/kg the first hour and 5 ml/kg the following two hours. Urinary samples were collected upon admission and the day after surgery. As surgery was initiated, urine was collected over the course of 4 h. Hereafter, another urine collection proceeded until the morning. Urine was analyzed for markers of AKI neutrophil gelatinase-associated lipocalin (NGAL) and kidney injury molecule-1 (KIM-1). Arterious and venous blood samples for measurements of pH and plasma electrolytes including chloride (p-Cl) were collected as surgery was initiated, at the end of surgery and the following morning.

**Results:**

IS induced an increase in p-Cl (111 ± 2 mmol/L after IS and 108 ± 3 after PL, *p* = 0.004) and a decrease in pH (7.39 ± 0.02 after IS and 7.43 ± 0.03 after PL, *p* = 0.001). Urinary NGAL excretion increased in both groups (ΔNGAL: 5.5 [4.1; 11.7] μg/mmol creatinine *p* = 0.004 after IS vs. 5.5 [2.1;9.4] μg/mmol creatinine after PL, *p* < 0.001). No difference was found between the groups (*p* = 0.839). Similarly, urinary KIM-1 excretion increased in both groups (ΔKIM-1: IS 115.8 [74.1; 156.2] ng/mmol creatinine, *p* < 0.001 vs. PL 152.4 [120.1; 307.9] ng/mmol creatinine, p < 0.001). No difference between the groups (*p* = 0.064).

FE_Na_ increased (1.08 ± 0.52% after IS and 1.66 ± 1.15% after PL, *p* = 0.032). ENaC excretion was different within groups (*p* = 0.019).

**Conclusion:**

A significantly higher plasma chloride and a lower pH was present in the group receiving isotonic saline. However, u-NGAL and u-KIM-1 increased significantly in both groups after surgery despite absence of changes in creatinine. These results indicate that surgery induced subclinical kidney injury. Also, the IS group had a delayed sodium excretion as compared to the PL group which may indicate that IS affects renal sodium excretion differently from PL.

**Trial registration:**

ClinicalTrials.gov Identifier: NCT02528448, 19/08/2015

## Introduction

Fluid treatment is widely used for maintaining cardiovascular stability in patients undergoing surgery, trauma, and critical disease. Both isotonic saline and balanced crystalloids are commonly used for fluid resuscitation [1, 2]. Isotonic saline has a high chloride content (154 mmol/L), whereas the balanced crystalloids have lower chloride contents but also differ in electrolyte composition. Ringer’s acetate, Ringer’s lactate and PlasmaLyte are all balanced crystalloids, and PlasmaLyte has the lowest chloride content (98 mmol/L). Infusion of fluids containing high chloride amounts are suspected to increase the need for renal replacement therapy. In several studies, isotonic saline induced hyperchloremic metabolic acidosis as opposed to fluids with a lower sodium and chloride content, especially when administered in high amounts [3–5]. Animal experiments have shown that hyperchloremic acidosis reduces renal blood flow (RBF) and induces kidney injury [3, 6–9]. High chloride concentrations during renal perfusion correlated with increased renal vasoconstriction and with declines in RBF and glomerular filtration rate (GFR) [7–9]. In addition, studies in healthy subjects comparing isotonic saline to fluids with lower sodium and chloride contents showed a decrease in RBF and GFR [8, 10].

Infusion of balanced IV solutions in patients admitted to an emergency department was associated with a lesser degree of acute kidney injury (AKI) than infusion of fluid solutions with higher chloride content [3, 5]. However, the importance of hyperchloremia and infusion of high chloride-containing solutions is still under much debate in the clinical setting [11–13]. In daily practice, traditional biomarkers such as plasma creatinine and GFR are used to estimate renal function. In case of AKI, changes in creatinine may require 24 h or more before being detectable. However, biomarkers such as neutrophil gelatinase-associated (NGAL) and kidney injury molecule-1 (KIM-1) have shown potential as acute indicators of AKI. Both are able to detect kidney injury and predict the risk of renal replacement therapy within hours [14–18].

We therefore hypothesized that fluids containing high amounts of chloride given as isotonic saline could induce hyperchloremic acidosis and cause subsequent changes in biomarkers of kidney injury. These effects may be detected by measuring renal function, urinary excretion of biomarkers of kidney injury, and plasma concentrations of vasoactive hormones.

We investigated this hypothesis in a randomized, double-blinded study, where patients undergoing primary uncemented hip replacement were given either isotonic saline (IS) or PlasmaLyte (PL) during the course of their surgery and recovery.

## Materials and methods

### Design

The study was a randomized, double-blinded study in 40 patients undergoing primary uncemented hip replacement.

### Recruitment

All patients were recruited from the Department of Orthopedic Surgery, Gødstrup Hospital, Holstebro; Denmark. Patients referred for elective hip arthroplasty were asked to participate. All patients were screened before participation. Screening examination included medical history, physical examination, office BP measurement, ECG, clinical biochemistry, and urine analysis. All patients were screened between August 2015 and February 2016.

### Patients

#### Inclusion criteria

Age > 18 years, patients undergoing primary uncemented hip replacement during spinal anesthesia.

#### Exclusion criteria

Blood donated within the past month, eGFR < 30 ml/min, pregnancy, nursing, diabetes mellitus type 1, or unwillingness to participate.

#### Withdrawal criteria

Estimated perioperative bleeding exceeding 1000 ml, blood transfusion, development of post-operative infection, reoperation, or withdrawal of consent.

### Outcomes

The main effect variable was u-NGAL. Other effect variables were u-KIM-1, free water clearance (C_H2O_)_,_ GFR, fractional excretion of sodium (FE_Na)_, fractional excretion of potassium (FE_K),_ fractional excretion of chloride (FE_Cl),_ u-albumin, urinary excretions of aquaporin-2 (u-AQP2), epithelial sodium channels (u-ENaC_γ_), Na-K-Cl cotransporter (u-NKCC2) and Na-Cl cotransporter (u-NCC), plasma concentration of renin (PRC), angiotensin II (p-AngII), aldosterone (p-Aldo) and vasopressin (p-AVP), arterial blood gas levels of pH, chloride (P-Cl), base excess (SBEc), and bicarbonate (P-HCO_3_^−^).

### Number of patients

With a significance level of 5% and a power of 80%, 16 patients in each group were needed to detect a 100 ng/mL difference in u-NGAL (SD 100 ng/mL). Due to expected drop-outs and complications during surgery, it was estimated that 20 subjects in each group should be included in the trial.

### Study medication

Patients were randomized to receive either isotonic saline (IS, 0.9% NaCl, 154 mmol/L chloride) or PlasmaLyte (PL, 98 mmol/L chloride). Both fluids were manufactured by Baxter A/S (Allerød, Denmark) and produced in 1000 ml Viaflo® bags (Baxter A/S). For blinding, each bag was concealed in identical white plastic. Three bags (identical fluid) were packed in boxes corresponding to each randomization number. The hospital pharmacy performed all blinding and packing.

The fluids were administered according to the guidelines from the Department of Anesthesiology, as a continuous intravenous infusion of 15 ml/kg/hour during the first hour of surgery and 5 ml/kg/hour the next two hours. To ensure hemodynamic stability due to blood loss, supplemental fluid could be administered to maintain a mean arterial pressure (MAP) of 70.

### Randomization

The hospital pharmacy generated the randomization list. The list was created in blocks of ten using the computer program “Randomization Generator”. Treatment assignment and allocation was concealed from clinicians, patients, and research staff until completion of the trial.

### Experimental procedure

#### Anesthetic procedure

Prior to surgery, patients received paracetamol 1000 mg orally. A peripheral venous catheter was positioned in a cubital vein for medication and blood sampling. Perioperatively, the patients were monitored with ECG, pulse oximeter and non-invasive blood pressure monitoring.

Prophylactic doses of cefuroxime 1.5 g and tranexamic acid 15 mg/kg (maximum 1 g) were administered. According to local guidelines during spinal anesthesia a urine bladder catheter was inserted in all patients. Spinal anesthesia was achieved with the patient in a lateral position according to the guidelines from the department of anesthesiology. If MAP decreased below 70 mmHg, additional intervention fluid or intermittent doses of phenylephrine 0.1 mg were administered. If arterial saturation decreased below 96%, additional oxygen was provided with a nasal cannula. During the course of the trial, patients were not allowed to receive ephedrine, dexamethasone, or nonsteroid anti-inflammatory drugs.

#### Urine and blood sampling

The day prior to surgery, all patients performed a 24-h urine collection (urine 1, baseline).

Upon admission, urinary spot samples were collected (urine 2, admission).

When surgery was initiated, urine was collected over the course of 4 h (urine 3, surgery) via the urinary catheter. Hereafter, another urine collection proceeded until the following morning at 8.00 am (urine 4, post-surgery). Every 4 h, urine bags were emptied and the urine stored at 5 °C.

After the urinary catheter had been removed, another urinary spot sample was collected before the patient was discharged (urine 5, discharge). Then, 12–14 days after surgery, patients provided a 24-h urine collection (urine 6, follow-up).

Urine samples were analyzed for u-NGAL, u-KIM-1, u-NKCC2, u-NCC, u-AQP2, u-ENaC_γ_, osmolality, u-albumin, u-chloride, u-sodium, u-potassium, and u-creatinine.

Arterial and venous blood samples were drawn right before anesthesia and intervention (baseline). After surgery, blood samples were drawn within the first 2 h after arrival in the recovery room and in the morning of the postoperative day. All blood samples were analyzed for measurements of pH, SBEc, P-HCO_3_^−^, and plasma concentrations of Cl^−^, Na^+^, K^+^, albumin, creatinine, Hemoglobin (Hgb), osmolality, PRC, P-AngII, P-Aldosterone and P-AVP.

#### Biochemical analyses

All urine and blood samples were kept frozen at − 80° or − 20 °C until assayed and were centrifuged again before analysis.

NGAL was determined by an enzyme-linked immunosorbent assay (ELISA) from Bioporto (Hellerup, Denmark) as previously described [19, 20]. Levels of minimal detection was 1.4 pg/ml. Variations were established as interassay max 8% and intraassay max 14%. KIM-1 was determined by an ELISA-kit (Quantijine ELISA) from R&D Systems (Minneapolis, USA) as previously described [20]. Levels of minimal detection was 3.0 pg/ml. Coefficients of variation were 7.8% (interassay) and 4.4% (intraassay). All samples were analyzed with kits from the same batch.

u-AQP2 and u-ENaC_γ_ were measured by radioimmunoassay as previously described [21–23]. Antibodies to synthetic peptides for ENaC_γ_ were raised in rabbits and the affinity purified as previously described [21, 22, 24]. For ENaCγ the levels of minimal detection was 48 pg per tube; coefficients of variation were 6.7% (intra-assay) and 14% (inter-assay). Similarly, antibodies for AQP2 were raised in rabbits [23]. These antibodies were raised to a synthetic peptide equivalent to the 15 COOH-terminal amino acids in human AQP2. Hereafter, an NH_2_-terminal cysteine was added for conjugation and affinity purification. The anti-AQP2 antibody was a gift from Søren Nielsen, The Water and Salt Research Center, Aarhus University, Denmark. For AQP2 the levels of minimal detection was 34 pg per tube; coefficients of variation were 5.9% (intra-assay) and 11.7% (inter-assay).

AngII and AVP were determined by radioimmunoassay as previously described [20, 24, 25]. The antibody against vasopressin was a gift from Professor Jacques Dürr, M.D., Tampa General Hospital, Tampa, Florida. NKCC2 and NCC was measured in urine by a radio radioimmunoassay as previously described [22, 26].

PRC and Aldosterone was determined by immunoradiometric assay as previously described [24]. Plasma and urine concentrations of sodium, potassium, chloride, albumin, and creatinine were routinely analysed at the Department of Clinical Biochemistry, Gødstrup Hospital, Denmark. To determine pH, SBEc, P-HCO_3_^−^, and plasma concentrations of Cl^−^, a blood gas analyzer (ABL800 Radiometer) was used at the Department of Anaesthesiology, Holstebro, Denmark.

#### Calculations

Free water clearance (C_H2O_) was calculated with use of the formula C_H2O_ = UO – C_osm_, where C_osm_ is osmolar clearance and UO is urinary output. Fractional excretions of sodium (FE_Na_), potassium (FE_K_), and chloride (FE_Cl_) were calculated with the formula FE_X_ = (X_u_ * V / X_p_)/GFR, where X_u_ and X_p_ are urine and plasma concentrations of X and V is urine flow in ml/min.

#### Statistics

Values showing normality are presented as means ± standard deviations (SD). If normality was not present, values are presented as medians with 25 and 75% percentiles in brackets.

Within group comparisons were performed with a paired t-test and an unpaired t-test was used for comparison between the groups. Nonparametric data with-in group were compared with a Wilcoxon signed-rank test and between groups with a Mann–Whitney U test. Comparison of two frequencies was done by chi-square test. Treatment effect was analyzed using a repeated measures general linear model (GLM). If data did not show normality, logarithmic transformation was performed prior to GLM. For the primary outcome variable statistical significance was defined as *p* < 0.05. All analyses on the secondary outcome variables were reviewed as hypothesis generating and there have not been made corrections for multiple testing. Statistical analyses were performed using PASW version 20.0.0 (SPSS Inc.; Chicago, IL, USA).

## Results

### Demographics

**Fig. 1:**
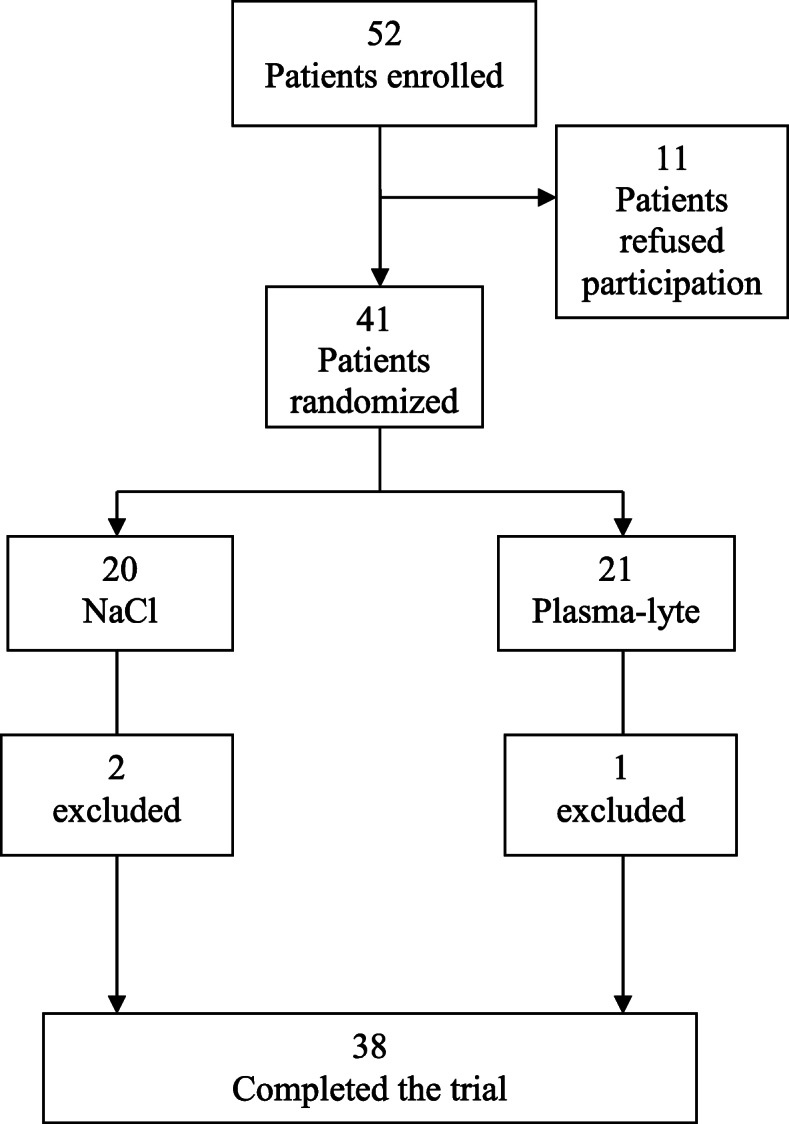
Flow chart showing the participant flow through screening, inclusion and completion of the trial

Fifty-two patients were screened for participation in the study. Eleven patients were unwilling to participate and 41 patients were randomized. Three patients were excluded due to perioperative bleeding above 1000 ml (*n* = 1, received IS), blood transfusion (n = 1, received IS) and suspected development of type 1 diabetes (n = 1, received PL). Thus, 18 patients who received IS and 20 patients who received PL were included in the analysis (Fig. 1). The two groups were comparable with regards to sex, age, body mass index, blood pressure, comorbidities, and screening biochemistry (Tables 1 and 2).

**Table 1:** Demographics

	PlasmaLyte	NaCl	P-value value
**Male % (n)**	66.7% (14)	70% (12)	0.825
**Age (years)**	64 ± 6	67 ± 7	0.304
**Height (cm)**	174 ± 9	176 ± 7	0.583
**Body weight (kg)**	88.7 ± 12.8	83.8 ± 15.8	0.301
**Body mass index, BMI (kg/m**^**2**^**)**	29.4 ± 5.3	27.0 ± 4.2	0.134
**Systolic blood pressure (mmhg)**	146 ± 16	146 ± 17	0.994
**Diastolic blood pressure (mmhg)**	89 ± 10	87 ± 11	0.449
**Heart rate (beats pr. min)**	67 ± 9	66 ± 12	0.902
**P-Creatinine (μmol/L)**	75 ± 12	74 ± 11	0.844
**eGFR (mL/min)**	82 ± 9	82 ± 9	0.854
**Diabetes type II % (n)**	10% (2)	0% (0)	0.168
**Hypertension % (n)**	50% (10)	27.7% (5)	0.162

Values are shown as means ± SD in brackets. Statistics are performed with Students t-test and frequency data with Chi-square test

**Table 2:** Urinary excretion of proteins from epithelial sodium channels, aquaporin-2 channels, Neutrophil gelatinase-associated lipocalin and kidney injury molecule-1

Period	Baseline (1 day before surgery)	Follow-up (14 days after surgery)
**U-Aqua (ng/mmol crea)**		
PlasmaLyte	194.3 [165.7;244.4]	225.7 [175.9;258.4]
Sodiumchloride	196.9 [169.3;245.7]	220.0 [191.5;294.8]*
**U-ENaC ((ng/mmol crea)**		
PlasmaLyte	116.8 ± 46.2	145.1 ± 46.9*
Sodiumchloride	120.6 ± 42.4	139.1 ± 42.2
**U-NCC (ng/mmol crea)**		
PlasmaLyte	105.1 ± 26.8	146.2 ± 46.4*
Sodiumchloride	94.9 ± 33.4	133.0 ± 53.3*
**U-NKCC (ng/mmol crea)**		
PlasmaLyte	154.2 ± 38.7	171.6 ± 57.4
Sodiumchloride	142.9 ± 33.5	155.5 ± 46.3
**U-NGAL (ng/mmol crea)**		
PlasmaLyte	1446 [1238;3161]	1840[1303;2679]
Sodiumchloride	1577[866;3403]	1658[961;2494]
**U-KIM (ng/mmol crea)**		
PlasmaLyte	109.4 ± 61.6^†^	134.5 ± 68.7*^†^
Sodiumchloride	73.7 ± 47.3	93.8 ± 59.9*
**U-NGAL (ng/mL)**		
PlasmaLyte	9.5 [8.0;13.5]	11.0 [7.3;13.0]
Sodiumchloride	10.5 [5.0;13.0]	9.5 [7.5;15.5]
**U-KIM (ng/mL)**		
PlasmaLyte	0.54 [0.38;0.68]	0.64 [0.44;0.93]*
Sodiumchloride	0.37 [0.19;0.57]	0.41 [0.24;0.80]

Neutrophil gelatinase-associated lipocalin (u-NGAL/creatinine) and kidney injury molecule-1 (u-KIM-1/ creatinine), γ-fraction of the epithelial sodium channel (u-ENaCγ/creatinine), aquaporin-2 (u-AQP-2/ creatinine). Values are shown as means ± SD in brackets or medians with 25 and 75 percentiles in brackets

Statistics are performed with unpaired t-test or Mann-Whitney test to test difference in response between treatments, ^†^ = *p* < 0.05. Paired t-test or Wilcoxon signed rank test was used to test statistical significant difference from baseline, * = *p* < 0.05

### Operative procedures

The two groups were comparable regarding duration of anesthesia, surgery and recovery, and length of hospital stay (Table 3). Blood loss and the amount of intravenous fluid given were the same in both groups. However, the total mass of chloride provided pr. Kg bodyweight to each patient, was higher in the IS group (0.09 g/kg in the PL group vs. 0.14 g/kg in the IS group, *p* < 0.001). No difference between the groups was observed in the number of patients receiving phenylephrine (6 in the PL group vs. 5 in the IS group, *p* = 0.8) or the average dose per patient (0.22 mg in the PL group vs. 0.46 mg in the IS group, *p* = 0.1).

**Table 3:** Perioperative Management

	PlasmaLyte	NaCl	P-value
**Time periods**			
**Duration of anesthesia (min)**	117 ± 29	122 ± 50	0.974
**Duration of the surgery (min)**	44 ± 17	42 ± 13	0.661
**Duration of the recovery period (min)**	205 ± 76	179 ± 68	0.323
**Length of hospital stay (hours)**	38 ± 11	34 ± 10	0.258
**Intervention fluid iv. (ml)**	2275 ± 377	2117 ± 437	0.239
**Total mass of chloride pr. patient (g)**	7.4 ± 1.5	12.4 ± 2.1	0.000
**Total mass of chloride pr. kg bodyweight (g/kg)**	0.09[0.09;0.10]	0.14[0.14;0.14]	0.000
**Blood loss during surgery (ml)**	324 ± 124	292 ± 152	0.503
**Patients needing phenylephrine, no. (%)**	6(33)	5(36)	0.880
**Phenylephrine dose pr. patient (mg)**	0.22 ± 0.12	0.46 ± 0.35	0.142

Values are shown as means ± SD in brackets or medians with 25 and 75 percentiles in brackets. Statistics are performed with students t-test or Mann-Whitney test and frequency data with a Chi-square test

### Arterial blood pH, SBEc, cHCO3 and levels of plasma chloride

**Fig. 2:**
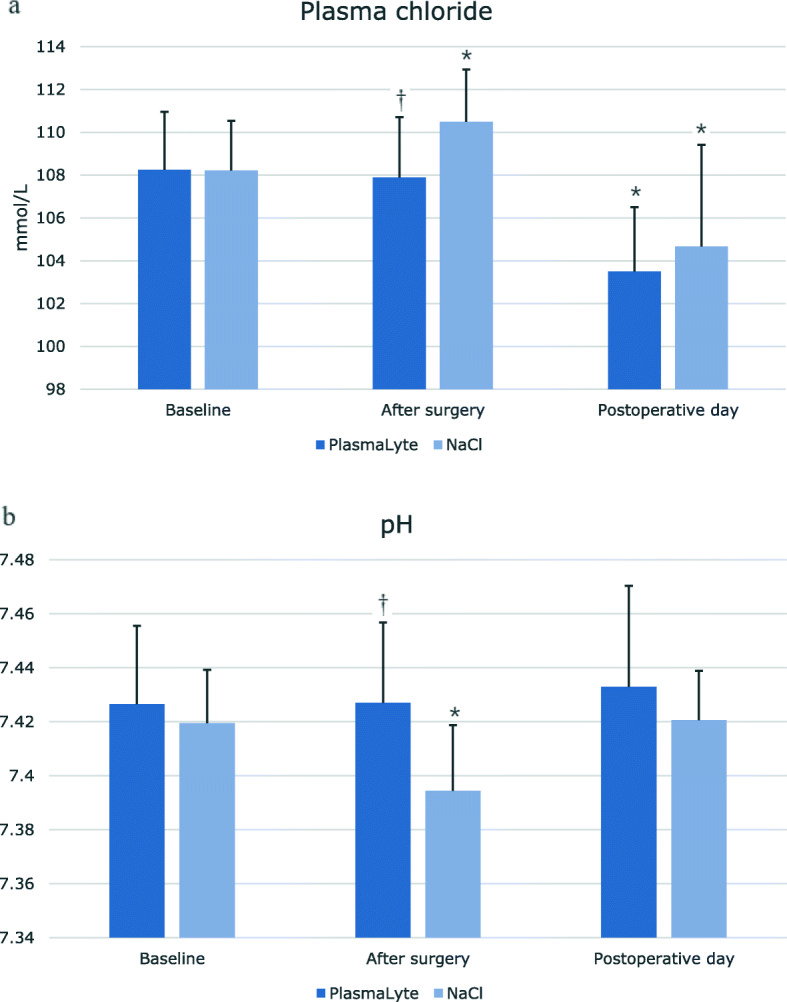
Effect of Isotonic saline vs. PlasmaLyte on arterial blood gas levels of chloride (**a**) and pH (**b**) in a double-blinded, placebo-controlled study of 38 patients. Blood samples were drawn before anesthesia (baseline), right after surgery (after surgery) and in the morning of the postoperative day (postoperative day). Values are shown as means ± SD. Statistics are performed with unparried t-test to test difference in response between treatments, ^†^ = *p* < 0.05. Paired t-test was used to test statistical significant difference from baseline, * = p < 0.05

The levels of arterial blood pH (Fig. 2a), SBEc and cHCO3 (Table 4) was significantly lower in the IS group after surgery, and plasma concentrations of Cl were significantly higher in the IS group as compared to the PL group after surgery (Fig. 2b). These differences were not present the day after surgery.

**Table 4:** Arterial Blood Gas

Period	Baseline	After surgery	Post-operative day
**cHCO3 (mmol/L)**			
PlasmaLyte	25.0 ± 1.6	24.9 ± 1.4^†^	25.7 ± 1.5*
Sodiumchloride	24.5 ± 1.3	22.9 ± 1.0*	24.8 ± 1.4
**SBEc (mmol/L)**			
PlasmaLyte	0.39 ± 2.00	0.33 ± 1.77^†^	1.34 ± 1.82*
Sodiumchloride	−0.93 ± 1.68	−2.02 ± 1.32*	0.34 ± 1.83

Arterial blood gas levels of bicarbonate (HCO3-) and base excess (SBEc)

Values are shown as means ± SD. Statistics are performed with unparried t-test or Mann-Whitney test to test difference in response between treatments, ^†^ = *p* < 0.05. Paired t-test or Wilcoxon signed rank test was used to test statistical significant difference from baseline, * = *p* < 0.05

### Urinary NGAL and KIM-1

**Fig. 3:**
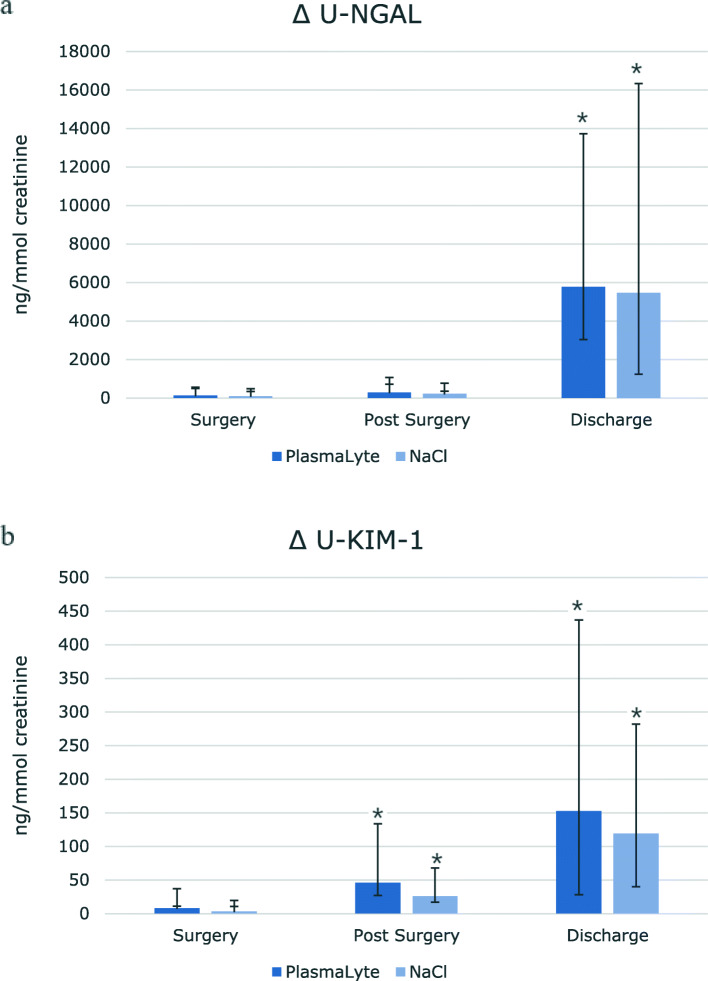
Change from baseline in urinary excretion rate of neutrophil gelatinase-associated lipocalin (NGAL) (**a**) and kidney injury molecule-1 (KIM-1) (**b**) in a double-blinded, placebo-controlled study of 38 patients. Values represent changes form baseline to surgery, post-surgery and discharge. The increase in u-NGAL and u-KIM-1 after isotonic saline and PlasmaLyte was observed in this period. Values are shown as medians with 25 and 75 percentiles in brackets. *P*-value represents difference from baseline, * = *p* < 0.05. Statistics are performed with a Wilcoxon signed rank test

u-NGAL and u-KIM-1 increased steadily in both groups during the study period (Table 5, Fig. 3). However, no difference in the increase between groups was seen. No significant difference in u-NGAL levels was detected, when comparing the 24 h urine collection at baseline and two weeks later (Table 2). u-KIM-1 levels were significantly higher in the PL group compared to the IS group both at baseline and two weeks later. Both groups had a significant increase in u-KIM-1 two weeks after surgery as compared to baseline values (Table 2).

**Table 5:** Urinary excretion of proteins from epithelial sodium channels, aquaporin-2 channels, Neutrophil gelatinase-associated lipocalin and kidney injury molecule-1

Period	Admission	Surgery	Post-surgery	Discharge	***P (GLM within)***
**U-NGAL (ug/mmol crea)**					
PlasmaLyte	1.03 [0.63;2.33]	1.03[0.79;1.68]	1.28 [0.81;2.68]	7.08* [5.66;13.18]	0.376
Sodiumchloride	0.78 [0.57;1.10]	0.88 [0.50;1.17]	1.01 [0.72;1.35]	7.97* [4.77;12.63]	
*P (GLM between)*	0.523				
**U-KIM (ng/mmol crea)**					
PlasmaLyte	97.0 [50.1;183.4]	92.4 [68.18;152.7]	179.5* [109.1;244.2]	301.0*[176.5;427.1]	0.324
Sodiumchloride	70.0 [37.9;117.7]	68.0 [45.9;134.6]	85.1* [67.7;167.8]	183.0* [125.5;313.2]	
*P (GLM between)*	0.116				
**U-NGAL (ng/mL)**					
PlasmaLyte	7.5[4.0;21.3]	5.0[2.0;8.0]*	14.0[7.3;31.8]*	72.0[43.3;158.5]*	0.644
Sodiumchloride	5.0[2.0;10.0]	3.5[2.0;6.0]	9.0[6.5;11.5]	142.0[66.3;196.3]*	
*P (GLM between)*	0.728				
**U-KIM (ng/mL)**					
PlasmaLyte	1.10[0.16;1.34]	0.46[0.09;0.68]	1.37[0.97;2.91]*	3.71[1.97;6.62]*	0.627
Sodiumchloride	0.41[0.12;0.86]	0.42[0.16;0.62]	0.75[0.43;1.61]*	2.48[1.12;4.91]*	
*P (GLM between)*	0.910				
**U-ENaC (ng/mmol crea)**					
PlasmaLyte	107.7 ± 45.7	137.0 ± 47.2^*^	132.2 ± 28.8*	233.8 ± 84.0*	0.019
Sodiumchloride	116.7 ± 52.0	195.3 ± 71.3*	141.5 ± 57.1	218.2 ± 52.8*	
*P (GLM between)*	0.222				
**U-Aqua (ng/mmol crea)**					
PlasmaLyte	180.2 [155.1;227.4]	263.9 [216.3;360.0]*	340.5 [275.3;455.7]*	357.1 [252.9;589.4]*	0.693
Sodiumchloride	192.6 [149.8;217.2]	334.9 [241.1;374.5]*	300.8 [229.4;393.9]*	416.9 [269.7;574.6]*	
*P (GLM between)*	0.972				
**U-NCC (ng/mmol crea)**					
PlasmaLyte	123.4 [99.4;137.4]	254.5 [174.5;320.7]*	156.7 [135.6;197.2]*	217.1 [193.1;288.0]*	0.321
Sodiumchloride	112.5 [77.5;156.8]	242.6 [201.3;271.0]*	191.4 [162.9;220.8]*	225.6 [185.6;283.2]*	
*P (GLM between)*	0.855				
**U-NKCC (ng/mmol crea)**					
PlasmaLyte	171.7 ± 70.9	338.4 ± 156.7*	179.1 ± 49.1	203.5 ± 80.0	0.304
Sodiumchloride	161.2 ± 58.2	283.9 ± 117.8*	186.3 ± 66.3	211.6 ± 106.4*	
*P (GLM between)*	0.566				

Neutrophil gelatinase-associated lipocalin (u-NGAL/creatinine) and kidney injury molecule-1 (u-KIM-1/ creatinine), γ-fraction of the epithelial sodium channel (u-ENaCγ/creatinine), aquaporin-2 (u-AQP-2/ creatinine). Values are shown as means ± SD or medians with 25 and 75 percentiles in brackets. Statistics are performed with a general linear model (GLM). Paired t-test or Wilcoxon signed rank test was used to test statistical significant difference from baseline with -in group, * = *p* < 0.05

### U-ENaC, u-AQP2, u-NCC and u-NKCC

The response in u-ENaC was not significantly different between groups. However, a difference was present within groups (*p* = 0.019). The PL group had a steady increase during the entire study period, whereas the IS group showed a more rapid increase during surgery and again at discharge (Table 5). u-AQP2 increased during surgery, post-surgery and at discharge in both groups. No difference was found between baseline and follow-up (Table 2). The differences were non-significant between the groups during the entire study (Table 5).

u-NCC excretion showed an increase as compared to baseline during the study for both groups. This increase was still present at follow-up (Table 2). But no significant difference in response was found. u-NKCC excretion showed a significant increase for both groups during surgery as compared to baseline. No difference was found for either group after surgery, but an increase was present for the saline group at discharge. No significant difference in response was found.

### Creatinine clearance, free water clearance and urine output

**Fig. 4:**
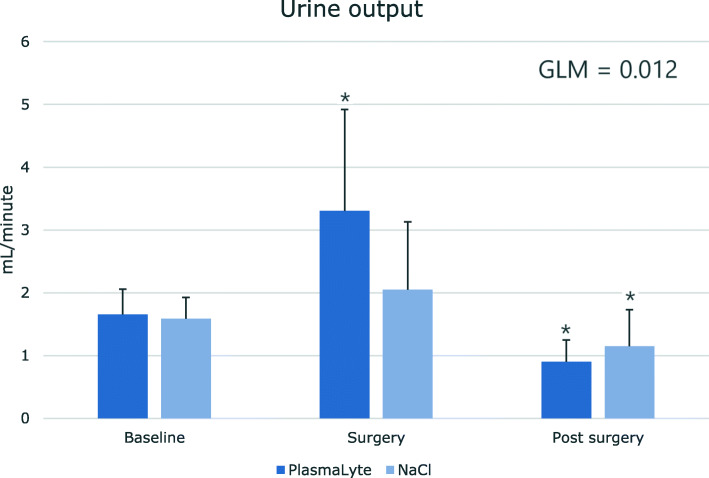
Effect of Isotonic saline vs. PlasmaLyte on urine output in a double-blinded, placebo-controlled study of 38 patients. Urine was collected as a 24-h urine collection (baseline) prior to surgery. As surgery was initiated urine was collected over the course of 4 h (surgery). Hereafter, another urine collection proceeded until the following morning at 8.00 am (post-surgery). Values are shown as means ± SD. Statistics are performed with unpaired t-test to test difference in response between treatments, ^†^ = p < 0.05. Paired t-test was used to test statistical significant difference from baseline, * = p < 0.05

During surgery, urine output (UO) was higher in the PL group (*P* = 0.012). But both groups showed a significant decrease post-surgery compared to baseline (Fig. 4). Post-surgery the PL group showed a significant decrease in CH_2_O as compared to baseline. No significant differences were found between the groups. Creatinine clearance (CrCl) was significantly higher in the PL group during both surgery and post-surgery compared to baseline. Yet, the IS group only had a significant increase in CrCl post-surgery compared to baseline. However, no significant differences were found between the groups (Table 6).

**Table 6:** GFR and tubular function

Period	Baseline	Surgery (0–4 h)	Post-surgery (4–24 h)	P-value (difference in response)
**C**_**H2O**_ **(mL/minute)**				
PlasmaLyte	−0.85 ± 0.66	−0.77 ± 1.21	−1.08 ± 0.43*	0.725
Sodiumchloride	−0.80 ± 0.72	−0.83 ± 0.70	−1.17 ± 0.49	
**Creatinine clearance (mL/minute)**				
PlasmaLyte	124.3 ± 31.4	147.4 ± 46.3*	137.3 ± 39.9*	0.362
Sodiumchloride	116.6 ± 29.3	129.2 ± 31.4	137.2 ± 29.4*	
**FE**_**K**_ **(%)**				
PlasmaLyte	11.6 ± 4.3	20.9 ± 8.6*	12.0 ± 6.2	0.002
Sodiumchloride	12.8 ± 3.3	16.2 ± 4.7*	10.7 ± 3.8*	
**FE**_**Cl**_ **(%)**				
PlasmaLyte	1.04 ± 0.80	1.45 ± 1.23	0.63 ± 0.46*	0.806
Sodiumchloride	1.05 ± 0.42	1.53 ± 0.69*	0.92 ± 0.49	

Free water clearance (CH2O), creatinine clearance, potassium (FEK) and Chloride (FECl). Values are shown as means ± SD or medians with 25 and 75 percentiles in brackets. Statistics are performed with a general linear model (GLM)

Paired t-test or Wilcoxon signed rank test was used to test statistical significant difference from baseline with -in group, * = *p* < 0.05

### FE_Na_, FE_K_ and FE_Cl_

FE_Na_ showed a significant increase for both groups during surgery compared to baseline (Fig. 6). The increase was more pronounced in the PL group (*p* = 0.032). No difference was found for either group on the postoperative day. FE_K_ showed a similar increase for both groups during surgery with a more pronounced increase in the PL group (*p* = 0.002). FE_K_ remained increased in the IS group on the postoperative day but no difference was found between groups (Table 6). FE_Cl_ showed a significant increase in the IS group after surgery. On the postoperative day a decrease was present in the PL group. However, the differences were non-significant between the groups during the entire study (Table 6).

### Vasoactive hormones in plasma

Table 7 shows the concentration of vasoactive hormones at baseline, after surgery and on the postoperative day. P-AVP showed a significant increase during the study as compared to baseline for both groups. The differences were non-significant between the groups. P-AngII decreased after surgery for the IS group but increased for the PL group on the postoperative day. No significant difference was found between groups. PRC were significantly higher in the PL group at baseline and after surgery as compared to the IS group, but no significant difference was found between groups on the postoperative day. P-Aldo was lower in both groups as compared to baseline after surgery and for the IS on the postoperative day. Also, no differences were found between groups.

**Table 7:** Vasoactive hormones

Period	Baseline	After surgery	Post-operative day	P-value (difference in response)
**p-AVP (pg/mL)**				
PlasmaLyte	0.3 [0.2;0.3]	0.5 [0.3;0.9]*	0.6 [0.4;0.8]*	0.398
Sodiumchloride	0.3 [0.3;0.4]	0.5 [0.3;1.4]*	0.5 [0.4;0.8]*	
**p-ANG2 (pg/mL)**				
PlasmaLyte	6.5 [3.3;9.0]	5.0 [3.3;8.8]	7.5 [4.3;12.0]*	0.174
Sodiumchloride	5.5 [3.0;7.5]	4.0 [2.8;5.0]*	6.5 [4.8;8.5]	
**p-Renin (pg/mL)**				
PlasmaLyte	11.2 [6.1;20.9]	9.9 [4.3;22.0]	11.9 [5.2;21.9]	0.540
Sodiumchloride	5.9 [3.3;7.2]	5.2 [3.2;7.0]*	7.6 [4.9;11.4]*	
**p-Aldo (pmol/L)**				
PlasmaLyte	113 [82;197]	48 [34;85]*	82 [53;142]	0.824
Sodiumchloride	129 [88;187]	50 [32;106]*	55 [43;96]*	

Plasma concentrations arginine vasopressin (p-AVP), angiotensin II (p-AngII), renin (PRC), and aldosterone (p-Aldo). Values are shown as means ± SD or medians with 25 and 75 percentiles in brackets. P-value represents probability of difference in response (response from baseline to infusion) between treatments. Paired t-test or Wilcoxon signed rank test was used to test statistical significant difference from baseline, * = *p* < 0.05

### P-Na, p-K, p-albumin, p-creatinine and hgb

Table 8 shows p-Na which decreased significantly in both groups on the post-operative day as compared to baseline. No significant difference was found between groups. After surgery p-K showed a significant decrease for the PL group as compared to the IS group. The IS group had a significant decrease compared to baseline both after surgery and on the postoperative day.

**Table 8:** Electrolytes

Period	Baseline	After surgery	Post-operative day	P-value (difference in response)
**p-Na (mmol/L)**				
PlasmaLyte	140 ± 2	140 ± 3	136 ± 3*	0.550
Sodiumchloride	139 ± 2	140 ± 2	135 ± 4*	
**p-K (mmol/L)**				
PlasmaLyte	3.9 ± 0.4	3.6 ± 0.3*	3.9 ± 0.4	0.346
Sodiumchloride	4.0 ± 0.3	3.8 ± 0.2 *	3.9 ± 0.2*	
**p-Albumin (g/L)**				
PlasmaLyte	40 ± 2	35 ± 2*	34 ± 2*	0.447
Sodiumchloride	39 ± 2	34 ± 2*	34 ± 2*	
**p-Creatinine (μmol/l)**				
PlasmaLyte	74 ± 14	66 ± 13*	66 ± 14*	0.264
Sodiumchloride	74 ± 10	68 ± 8*	65 ± 9*	
**p-Hgb (mmol/L)**				
PlasmaLyte	8.7 ± 0.6	7.9 ± 0.7*	7.4 ± 0.4*	0.931
Sodiumchloride	8.6 ± 0.6	7.8 ± 0.5*	7.4 ± 0.8*	

Plasma concentrations of sodium (p-Na), potassium (p-K), albumin (p-Albumin), creatinine (p-creatinine) and Hemoglobin (p-Hgb). Values are shown as means ± SD or medians with 25 and 75 percentiles in brackets. P-value represents probability of difference in response (response from baseline to infusion) between treatments. Paired t-test or Wilcoxon signed rank test was used to test statistical significant difference from baseline, * = *p* < 0.05

p-albumin, p-creatinine and hgb decreased in both groups, with no difference between groups.

## Discussion

This study aimed to investigate whether IS infusion during elective surgery causes kidney injury, indicated by an increase in urinary markers of kidney injury: u-NGAL and u-KIM-1. In this randomized, double-blinded study, 0.9% NaCl (IS) infusion was compared with the balanced IV solution PlasmaLyte (PL) in patients with normal renal function. Both the IS and PL group had a marked increase in u-NGAL and u-KIM-1 excretion on the postoperative day with no difference between the two groups and our evidence does not support the hypothesis that IS causes more kidney injury than a balanced solution.

Animal studies have shown that high chloride-containing fluids induce an increased inflammatory response, coagulopathy, decreased renal perfusion and AKI [27]. In humans, evidence to support that chloride contents in intravenous solutions affect clinical outcome is controversial [27]. Several studies comparing IS with balanced solutions, suggest that these solutions may reduce the risk of AKI, the use of renal replacement therapy, coagulopathy, inflammation, the use of blood transfusion and mortality [28–34]. In contrast, several other clinical trials have shown no benefit to the use of the same balanced solutions [35–39]. Our trial does not support that high chloride-containing solutions causes more kidney injury than balanced solutions. However, our results may have been confounded by several factors.

Primarily, the response to IS infusion may be hidden within the response of the surgical procedure. A previous study by Mose et al. investigated the effect of hypertonic saline in healthy young subjects, without surgery [20]. They found a small but significant increase in both u-NGAL and u-KIM-1. In our trial, we found a high increase in biomarkers for both groups. When comparing our results to Mose et al. it is possible that the major part of the biomarker response is due to the surgical procedure. Thus, making it probable that the presumed effect of the hyperchloremia in the IS group have been hidden by the surgical procedure.

Another study by Soliman et al. have investigated the development of AKI in 285 chronic kidney disease (CKD) patients (stage 3–5) after total joint arthroplasty and found a 30% incidence of AKI [40]. However, in our study we only included patients with normal renal function (eGFR> 60 ml/min). Since, CKD increases the risk of developing AKI after surgery our results are difficult to compare.

In addition, a recent study conducted by Maheshwari et al. investigated 8616 mainly healthy surgical patients receiving either Lactated Ringer’s solution or IS. Interestingly, the study also found no difference in postoperative complications, in-hospital mortality, or postoperative AKI between the two groups [41]. However, In Maheshwari et al. patients received modest amounts of fluid (1900 ml) which may not be sufficient to induce kidney injury [41].

In accordance, a large trial compared PL and IS in 4 intensive care units found no difference in the risk of AKI between the two solutions [31]. The two groups also received similar volumes of fluid with a median of 2000 mL [31]. In our study a comparable amount of fluid was used in each group (Table 2). In a clinical setting 2000 ml of fluid is a lesser amount when treating critically ill patients. Other studies with similar or smaller amounts of fluid have shown that IS increases the need of renal replacement therapy among the critically ill [42–44]. In summary, these studies combined with our findings may suggest that modest volumes of IS in previously healthy patients do not result in further kidney injury than balanced solutions. Contrary, infusion of IS in critically ill patients may result in increased risk of AKI and the need of renal replacement therapy [5, 34, 42–44].

In the last decade, several studies on new biomarkers have been published in the search of a new diagnostic tool for AKI. In our study, two biomarkers of kidney injury were measured, i.e. NGAL and KIM-1. These have been related to increased risk of renal replacement therapy and CKD in patients with AKI [14–18]. Yet, NGAL is related to neutrophil leucocytes, and bacteremia can elevate excretion [45]. Accordingly, NGAL’s diagnostic properties can be affected in heterogeneous patient populations as the critically ill [46]. In our study, we investigated a similar patient population of mainly healthy patients, suggesting NGAL as a reasonable choice of biomarker. Interestingly, another study measuring NGAL and KIM-1 in response to AKI found an AUC-ROC value of 0.938, a sensitivity of 90% and a specificity of 100% when combining the two biomarkers [47]. This finding suggests, that the combination of these two biomarkers better predicts kidney injury than using only a single biomarker.

It is also possible that IS and PL share similar properties to induce kidney injury. In the above we have discussed the presumed nephrotoxicity of IS, but our results could also suggest that IS and PL are equally able to induce kidney injury. PL is said to be a balanced fluid, because its composition resembles plasma [48]. In human plasma the normal chloride content is between 98 and 106 mmol/L [48]. IS has a chloride content of 154 mmol/L and PL a chloride content of 98 mmol/L [48]. As mentioned, several studies have shown IS to induce hyperchloremic acidosis when compared to a balanced solution [3, 4, 6, 8, 35]. Volta et al. have investigated 40 surgical patients and found significantly lower levels of NGAL excretion in patients receiving Ringer’s acetate as compared to IS [44]. However, in our study investigating 38 patients in a similar setting, we found no difference in biomarker excretion. Additionally, an experimental study investigating 4 different crystalloids in an animal model of hypovolemic shock found the worst survival rate when using PL as compared to IS and lactated solutions [49]. Several studies have found that balanced solutions appear to be more physiological than IS and have found IS to result in hyperchloremic acidosis and adverse renal outcomes in patients [3, 8, 42]. Nevertheless, further evidence proving balanced solutions to result in improved patient outcomes is needed.

**Fig. 5:**
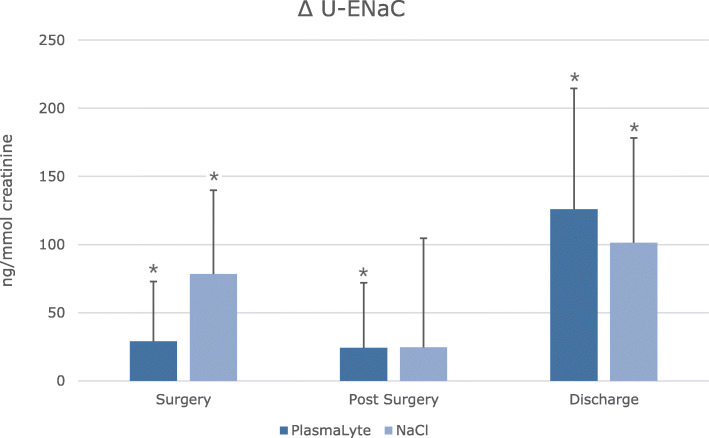
Change from baseline in urinary excretion rate of ENaC_γ_ in a double-blinded, placebo-controlled study of 38 patients. Values represent changes form baseline to surgery, post-surgery and discharge. Values are shown as means ± SD. Data are shown as means ± SD. Statistics are performed with unpaired t-test to test difference in response between treatments, ^†^ = p < 0.05. Paired t-test was used to test statistical significant difference from baseline, * = p < 0.05

**Fig. 6:**
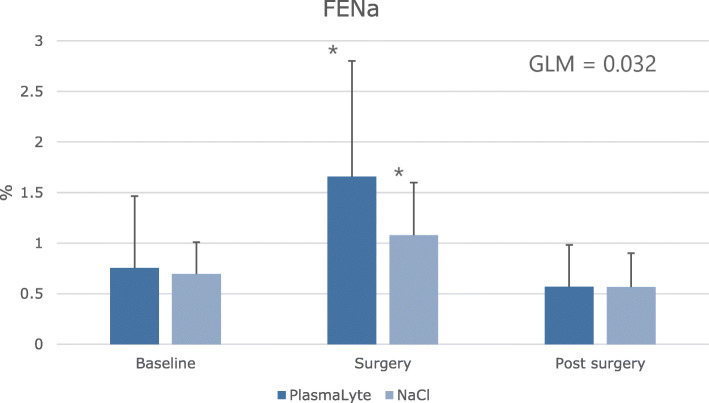
Effect of Isotonic saline vs. PlasmaLyte on fractional excretion of sodium (FENa) in a double-blinded, placebo-controlled study of 38 patients. Urine was collected as a 24-h urine collection (baseline) prior to surgery. As surgery was initiated urine was collected over the course of 4 h (surgery). Hereafter, another urine collection proceeded until the following morning at 8.00 am (post-surgery). Values are shown as means ± SD Data are shown as means ± SD. Statistics are performed with unpaired t-test to test difference in response between treatments, ^†^ = p < 0.05. Paired t-test was used to test statistical significant difference from baseline, * = *p* < 0.05

During surgery, creatinine clearance (CrCl) increased significantly in the PL group, as opposed to the IS group. It is well known that sodium loading increases CrCl, however PL and IS share a similar sodium content (IS 154 mmol/l and PL 140 mmol/l). A substantial difference between the two fluids is the chloride content (IS 154 mmol/l and PL 98 mmol/l). A high chloride amount can cause a decrease in RBF and explain the lower CrCL and UO in the IS group [8, 10]. These findings may suggest that the use of PL is associated with an augmented ability to handle an acute water and salt load. As a measure of tubular function, we investigated tubular transport proteins (AQP2, ENaC, NCC and NKCC2) which increased in both groups after surgery. u-ENaCγ had a significantly different response between the two groups (Table 5), with a higher u-ENaCγ excretion during surgery in the IS group (Fig. 5). A previous study conducted by Jensen et al. investigated the renal response on sodium and water transport of heathy adults receiving glucose, isotonic or hypertonic saline [21]. Their results were similar to ours, with an augmented u-AQP2 and u-ENaCγ response to saline infusion [21]. The increase in ENaCγ could be explained by a decrease in sodium absorption in the proximal part of the nephron, which would be compensated by the distal part. However, the above do not explain the difference in ENaCγ excretion between the two groups. The groups did not differ with regard to vasoactive hormones or AVP and the ENaCγ excretion seems independent of aldosterone (Table 7). Furthermore, when considering the remaining tubular transport proteins: AQP2, NCC and NKCC2 no differences were present between the two groups. This finding could imply that IS is associated with increased sodium retention during an acute water and salt load as compared to PL. Though, the increase in ENaCγ does not necessarily imply increased transcription and activity, but could be due to alterations in synthesis or degradation [50]. The augmented u-ENaCγ excretion as measure of increased sodium retention are substantiated by the decreased FENa (Fig. 6) and FEK (Table 6) in the IS group as compared to the PL group. The above, may indicate that isotonic saline can affect the kidneys response to an acute water and salt load.

In the current study, the power calculation (significance level of 5% and a power of 80%) was based on a rather large effect size (100 ng/mL) to ensure clinical relevance. The sample size (38 patients) was based on u-NGAL as the primary outcome measure. Furthermore, to explore the mechanisms of the intervention and differing responses, this study also examined several secondary outcome measures. However, the secondary outcome measures serves the purpose of a mechanistic hypothesis, which might increase the risk of committing a type 2 error. Although, the assessments of the secondary outcome measures can be considered as theoretical, these measures are of interest. Our results therefore should be viewed as hypothesis generating, hopefully contributing to further research in this area.

The major strength of this study is the randomized, double-blinded design. In both groups study conditions were comparable with regards to operative procedures, anesthesia and recovery period. To assess renal function a 24-h urine collection was performed both before admission and two weeks after surgery. The trial did not evaluate the long-term effect (> 14 days after surgery) of isotonic saline as compared to PL on renal function. However, a delayed kidney injury would be considered unlikely when no signs of renal impairment were seen 14 days after surgery.

## Conclusion

In conclusion, this randomized, double-blinded study did find significantly higher levels of chloride and lower pH in the group receiving isotonic saline after intraoperative infusion of isotonic saline during hip arthroplasty. However, we found no difference between groups in the excretion of NGAL or KIM-1 and their responses to fluid infusion. Both NGAL and KIM-1 were significantly increased in both groups after surgery, despite absence of rises in creatinine. These results may indicate that surgery induced subclinical kidney injury. Our results also showed a delayed excretion of sodium in the IS group as compared to the PL group which may indicate that IS affects kidney function and sodium balance differently, compared to PL.

## Data Availability

The datasets used and analyzed during the current study are available from the corresponding author on reasonable request.

## Abbreviations

AKI: Acute kidney injury
AQP2: Aquaporin-2
AUC-ROC: Area under a receiver operating characteristic curve
BP: Blood pressure
C_H2O_: Free water clearance
CKD: Chronic kidney disease
Cl^−^: Chloride
CrCl: Creatinine clearance
ECG: Electrocardiogram
eGFR: Estimated glomerular filtration rate
ELISA: Enzyme-linked immunosorbent assay
ENaC: Epithelial sodium channels
FE_Cl_: Fractional excretion of chloride
FE_K_: Fractional excretion of potassium
FE_Na_: Fractional excretion of sodium
GFR: Glomerular filtration rate
GLM: General linear model
Hgb: Hemoglobin
IS: Isotonic saline
K^+^: Postassium
KIM-1: Kidney injury molecule-1
MAP: Mean arterial pressure
Na^+^: Sodium
NCC: Na-Cl cotransporter
NGAL: Neutrophil gelatinase-associated lipocalin
NKCC2: Na-K-Cl cotransporter
p-Aldo: Plasma aldosterone
p-AngII: Plasma angiotensin II
p-AVP: Plasma vasopressin
P-HCO_3_^−^: Bicarbonate
p-K: Plasma potassium
p-Na: Plasma sodium
PL: PlasmaLyte
p-Cl: Plasma chloride
PRC: Plasma concentration of renin
SBEc: Base excess
SD: Standard deviation
u-AQP2: Urinary excretions of aquaporin-2
u-ENaC_γ_: Urinary excretions of epithelial sodium channels
u-NCC: Urinary excretions of Na-Cl cotransporter
u-NKCC2: Urinary excretions of Na-K-Cl cotransporter
UO: Urine output
